# A Delphi consensus on clinical features, diagnosis and treatment of major depressive disorder patients with anhedonia amongst psychiatrists in the Asia-Pacific

**DOI:** 10.3389/fpsyt.2024.1338063

**Published:** 2024-02-23

**Authors:** Calvin Cheng, Keira Herr, Hong Jin Jeon, Tadafumi Kato, Chee H. Ng, Yen Kuang Yang, Ling Zhang

**Affiliations:** ^1^ Department of Psychiatry, University of Hong Kong, Hong Kong SAR, China; ^2^ Janssen Medical Affairs Asia Pacific, Singapore, Singapore; ^3^ Department of Psychiatry, Depression Center, Samsung Medical Center, Sungkyunkwan University School of Medicine, Seoul, Republic of Korea; ^4^ Department of Psychiatry & Behavioral Science, Juntendo University Graduate School of Medicine, Tokyo, Japan; ^5^ The Melbourne Clinic, Department of Psychiatry, University of Melbourne, Melbourne, VIC, Australia; ^6^ Department of Psychiatry, National Cheng Kung University Hospital, Tainan, Taiwan; ^7^ National Clinical Research Center for Mental Disorders & Mood Disorders Center, Beijing Anding Hospital, Capital Medical University, Beijing, China

**Keywords:** anhedonia, major depressive disorder, DSM-5, Delphi consensus, Asia Pacific

## Abstract

**Background:**

Anhedonia, a core diagnostic feature for major depressive disorder (MDD), is defined as the loss of pleasure and interest in daily activities. Its prevalence in MDD patients vary from 35 to 70%. Anhedonia in MDD negatively impacts functioning and is associated with treatment resistance and poorer prognosis for various clinical outcomes. Owing to its complexity, there remains considerable heterogeneity in the conceptualization, diagnosis and clinical management of anhedonia in MDD.

**Methods:**

This modified Delphi panel was conducted to elicit expert opinion and establish consensus on concepts relating to clinical features, diagnosis and treatment of MDD with anhedonia (MDDwA) amongst psychiatrists in the Asia-Pacific region. Seven themes were covered. A three-stage process was adopted for consensus generation (two online survey rounds, followed by a moderated consensus meeting). Statements were developed based on a literature review and input from a steering committee of six regional experts. The panel included 12 psychiatrists practicing in Australia, China, Hong Kong, Japan, South Korea and Taiwan with ≥5 years of specialist clinical experience, including assessment or management of patients with MDDwA.

**Results:**

Overall, consensus was achieved (median ≥8) on 89/103 statements (86%). About half of the statements (55/103, 53%) achieved consensus in Round 1, and 29/36 modified statements achieved consensus in Round 2. At the moderated consensus meeting, five modified statements were discussed by the steering committee and consensus was achieved on all statements (5/5). The findings highlighted a lack of clear and practical methods in clinical practice for assessing anhedonia in MDD patients and limited physician awareness of anhedonia in Asia-Pacific.

**Conclusion:**

Insights from this Delphi consensus provide a reference point for psychiatrists in Asia-Pacific to optimize their strategies for personalized diagnosis and management of patients with MDDwA. Identification of distinct and clinically relevant subtypes in MDD may be valuable for guiding personalized diagnosis and management approaches, including type-specific therapies.

## 1 Introduction

Major depressive disorder (MDD) affects a substantial and growing number of individuals worldwide (estimated 280 million people) and is a leading cause of morbidity and disability (1–3). MDD imposes a substantial burden on individuals, adversely affecting their daily functioning and quality of life (4–6). According to the Diagnostic and Statistical Manual of Mental Disorders (5^th^ edition; DSM-5), a diagnosis of MDD requires at least five depressive symptoms, one of which must be depressed mood or anhedonia (the loss of pleasure or interest in daily activities), associated with a change from previous functioning (7). Although anhedonia is considered a core diagnostic feature of MDD, there is considerable heterogeneity in how the concept is defined and operationalized and consequently in how it is diagnosed and managed (8, 9). Anhedonia in MDD has been described as a symptom complex that encompasses overlapping concepts and various deficits in reward processing and positive affect. This presents notable challenges for diagnosis; indeed, estimates in the literature of anhedonia prevalence in MDD patients range from 35–70% (10, 11).

The impact on patients and clinical burden associated with anhedonia in MDD are substantial but often overlooked. Notably, anhedonia adversely affects psychosocial functioning, which can reduce the likelihood of achieving remission in MDD patients (12). In addition, anhedonia is a predictor of poor antidepressant treatment outcomes (13) and poorer prognosis for various clinical outcomes, regardless of depression severity (9). Moreover, a recent study found that in patients with mood disorders, including MDD, anhedonia was strongly correlated with quality of life (QoL): more severe anhedonia was associated with poorer physical and mental health-related QoL, and reduced life enjoyment and satisfaction (14). However, anhedonia in MDD often proves difficult to treat, as most conventional MDD pharmacotherapies do not adequately address anhedonia symptoms. Some commonly used first-line selective serotonin reuptake inhibitors (SSRIs) may even have a negative impact as they are known to blunt responses to rewarding stimuli (15).

Given the importance of anhedonia in MDD diagnosis and treatment, there has been increased interest and progress in reconceptualizing and assessing anhedonia, with the aim of addressing practical diagnostic and management challenges. Even so, findings from preclinical and clinical studies have been inconsistent, hindering translation to clinical practice (8, 16). Current anhedonia-specific assessment tools (symptom scales and behavioral tasks) reflect a variety of conceptualizations [reviewed in (8)]. For example, the Snaith-Hamilton Pleasure Scale (SHAPS), Fawcett-Clark Pleasure Capacity Scale, and Dimensional Anhedonia Rating Scale (DARS) assess different dimensions of anhedonia, and not all are specific to depression (17–19). Other depression-specific instruments for assessing symptom severity, such as the Hamilton Depression Rating Scale (HAM-D), include only a limited number of items for anhedonia. It has been proposed that the use of symptom scales alongside behavioral methods and biomarkers (if these can be identified) would enable a comprehensive assessment of the range of possible deficits associated with anhedonia in MDD (8, 16). On the other hand, it remains unclear how these insights are best applied in clinical settings. Nevertheless, defining distinct subtypes of MDD (e.g., based on biological variables and neuroimaging alongside clinical symptoms) is viewed as a promising strategy to improve diagnosis and management, as well as to predict the disease course (20, 21).

Issues such as diagnostic and symptom heterogeneity, along with the paucity of suitable clinical tools and specific guidelines, may contribute to substantial regional variability in the diagnosis and management of MDD with anhedonia (MDDwA). A modified Delphi panel study was therefore conducted to elicit expert opinion amongst psychiatrists in the Asia-Pacific region and establish consensus on concepts relating to clinical features, diagnosis, and treatment of MDDwA.

## 2 Methods

### 2.1 Modified Delphi process for consensus generation

**Figure 1:**
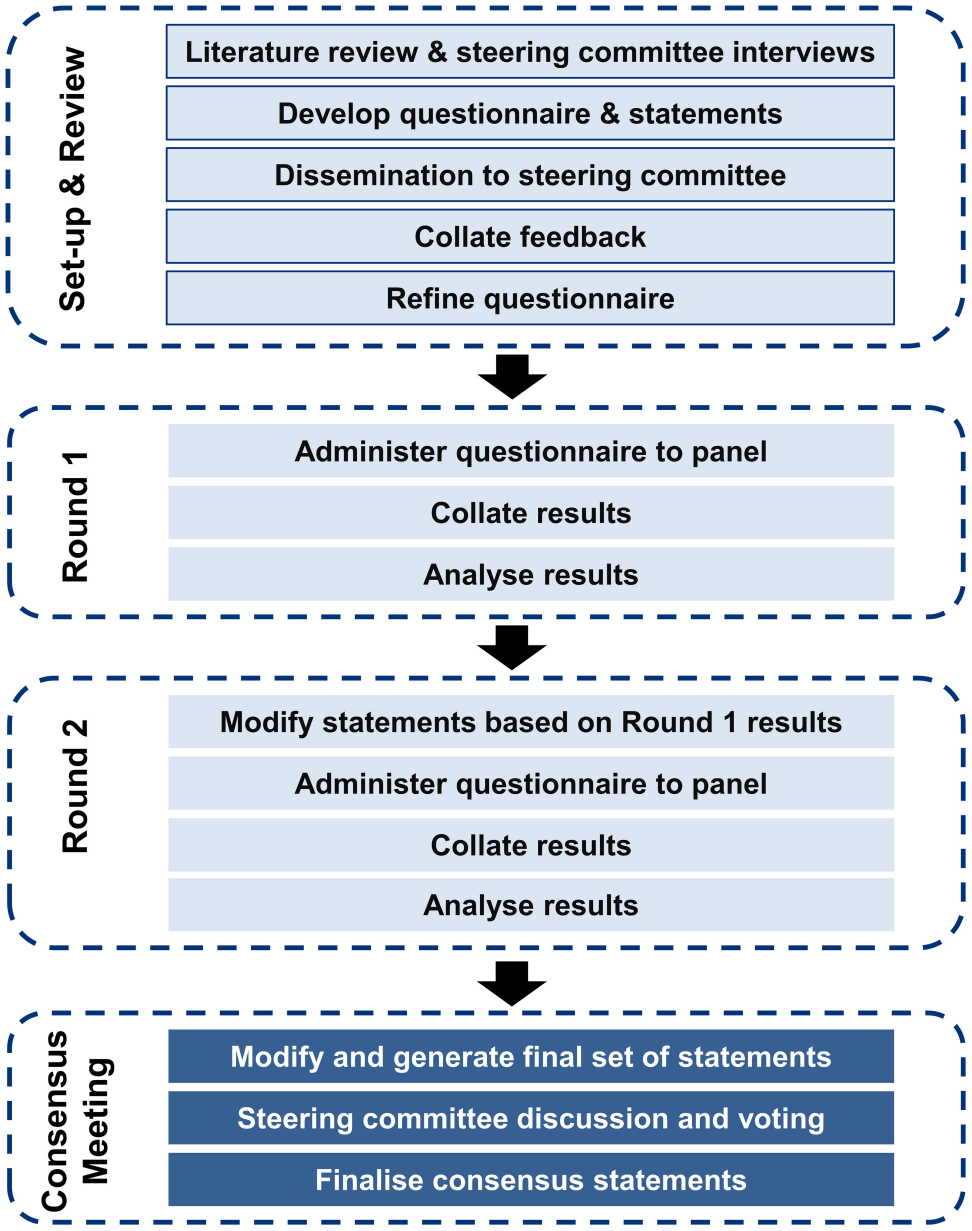
Modified Delphi process for consensus generation.

The Delphi method is a systematic and structured approach for identifying the consensus views of a group of experts in areas where the available evidence is limited or conflicting. An iterative process is followed, involving multiple rounds of surveys completed independently by a panel of experts. After each round, the experts review the collated anonymized responses and may revise their responses until consensus is reached based on pre-defined criteria. This method has been used to generate consensus among experts on topics such as the definition and assessment of treatment-resistant depression and culturally-relevant adaptation of mental health first-aid guidelines (22, 23). A modified Delphi panel study was conducted to generate consensus on research questions related to anhedonia in MDD among psychiatrists practicing in the Asia-Pacific region. The study involved four phases: panel identification and recruitment, questionnaire development, survey data collection and analysis (two rounds), followed by a moderated meeting to finalize the consensus statements. **Figure 1** illustrates the modified Delphi process for consensus generation. A steering committee of six regional experts (the authors of this work) provided oversight and input on study design, questionnaire development, and panel eligibility criteria and/or nomination. Details of the modified Delphi process are provided in the **Supplementary Methods**.

### 2.2 Identification and recruitment of Delphi panel participants

The panel comprised 12 experienced psychiatrists currently practicing in Australia, China, Hong Kong, Japan, South Korea, or Taiwan. Psychiatrists meeting the following criteria were identified: currently involved in diagnosis, treatment and management of MDD patients with anhedonia; at least five years of experience and expertise in diagnosing, treating and managing MDD patients with anhedonia; average monthly caseload of at least 20 patients suspected or confirmed to have MDD; average monthly caseload of at least ten patients suspected or confirmed to have MDD with anhedonia; possess experience of journal/conference authorship, academic, guideline development or clinical leadership related to MDDwA. Of 293 potential participants identified across the six countries/territories according to the eligibility criteria, 18 were found to meet these criteria. Only participants who were able and willing to complete both rounds of the study were recruited. The panel included 12 psychiatrists (two per country/territory) in total.

### 2.3 Survey questionnaire development and consensus classification

A targeted literature review was conducted on the epidemiology and best-practice recommendations for the diagnosis and management of MDDwA in the Asia-Pacific region. The literature search strategy (**Supplementary Table 1**) was designed to identify publications on these topics: current opinion on the definition of anhedonia in MDD or diagnosis of patients with MDDwA; current treatment options for patients with MDDwA; opinions on the diagnosis and treatment of patients with MDDwA; clinical guidelines specific to MDDwA. The information retrieved and literature gaps identified (**Supplementary Table 2**) were used to guide questionnaire development for the Round 1 survey.

A set of statements for the Round 1 survey were developed based on the literature review and input from the steering committee on clinical accuracy, comprehensibility, and feasibility for use in the online survey. The statements covered themes related to MDDwA, including: prevalence; risk factors; clinical definitions; diagnosis; treatment and management; disease impact and physician perspectives on novel therapy options. Each statement was to be scored by panel participants according to a 9-point Likert scale, which quantifies how strongly the participant agrees/disagrees with the statement (1–3, strongly disagree; 4–6, neither agree or disagree; 7–9, strongly agree). A free-text response/rationale was requested whenever a participant gave a statement a rating below 7 (neither agree or disagree; strongly disagree). For multiple-option questions, participants were required to select any options they considered applicable. For two such options, ‘None of the above (please provide rationale)’ and ‘Others (please specify)’, participants were required to provide input as free text. Responses to open-ended questions were also provided as free text. The free-text responses collected in both survey rounds were used to guide edits to improve clarity, merge or modify statements, and formulate additional statements or questions for consideration.

### 2.4 Data collection and analysis

The process of consensus generation is summarized in **Figure 1**. After providing consent to participate in the study, each panelist received a personalized email link to a password-protected electronic survey platform. Panelists were asked to complete the surveys independently and remained blinded to the identities of other panelists and the steering committee throughout the process. For both Rounds 1 and 2, panelists were asked to provide their responses within one week, with email reminders as needed. Responses from all 12 panelists were required in order to proceed with Round 2.

Descriptive statistics were used to summarize and analyze the response data (e.g., mean, median, standard deviation [SD] and interquartile range [IQR] of statement ratings, counts and proportions of participants selecting each response option). The consensus classification for each statement was determined according to the pre-defined criteria in **Table 1**.

**Table 1:** Criteria for consensus classification.

Classification	Threshold applied			
	Agreement [9-point Likert scale^1,2^]		Multiple option	Free-text response [% of panelists with the same opinion(s)/perspective(s)]
	% rated as 7–9	Median	% selected option	
Consensus	≥80%	≥8	≥80%	≥80%
Moderate consensus	≥70% but <80%	≥7 but <8	≥70% but <80%	≥70%
Nearing consensus	≥60% but <70%	≥6 but <7	≥60% but <70%	≥60%
No consensus	<60%	N/A	<60%	<60%

^1^Quantifies extent of agreement with the statement on a scale from 1–9 (1–3, strongly disagree; 4–6, neither agree or disagree; 7–9, strongly agree).

^2^For conflicts between percentage rating and median score, the median score was used as the main determinant of consensus classification, while the percentage rating guided the analysis and extent of question modification.

N/A, not applicable.

In Round 1, statements that achieved consensus (median score ≥8) were included in the final list of consensus statements. Statements with a median rating of ≥6 but <8 were modified for presentation in Round 2 based on input from the panel, which was subsequently validated by the steering committee. Statements that did not achieve consensus (median score <6) were removed from the set of consensus statements. However, depending on clinical importance, selected few statements were retained as key insights for discussion during the moderated consensus meeting. In the Round 2 survey, panelists were shown the anonymized and consolidated Round 1 results and were asked to score the modified statements derived from Round 1.

### 2.5 Moderated consensus meeting

After both survey rounds were completed, a moderated virtual meeting was held to facilitate interactive discussion within the steering committee to ratify the findings and identify areas of consensus and other issues of clinical importance (**Figure 1**). Consolidated responses and analyses from Rounds 1 and 2 were presented to allow the steering committee to vote on statements that did not achieve consensus in Round 2. Voting utilized real-time anonymous electronic polling functionalities. A final report summarizing the objectives, methodology, final consensus statements, and conclusions was developed and disseminated to the steering committee and the expert panel.

## 3 Results and discussion

**Figure 2:**
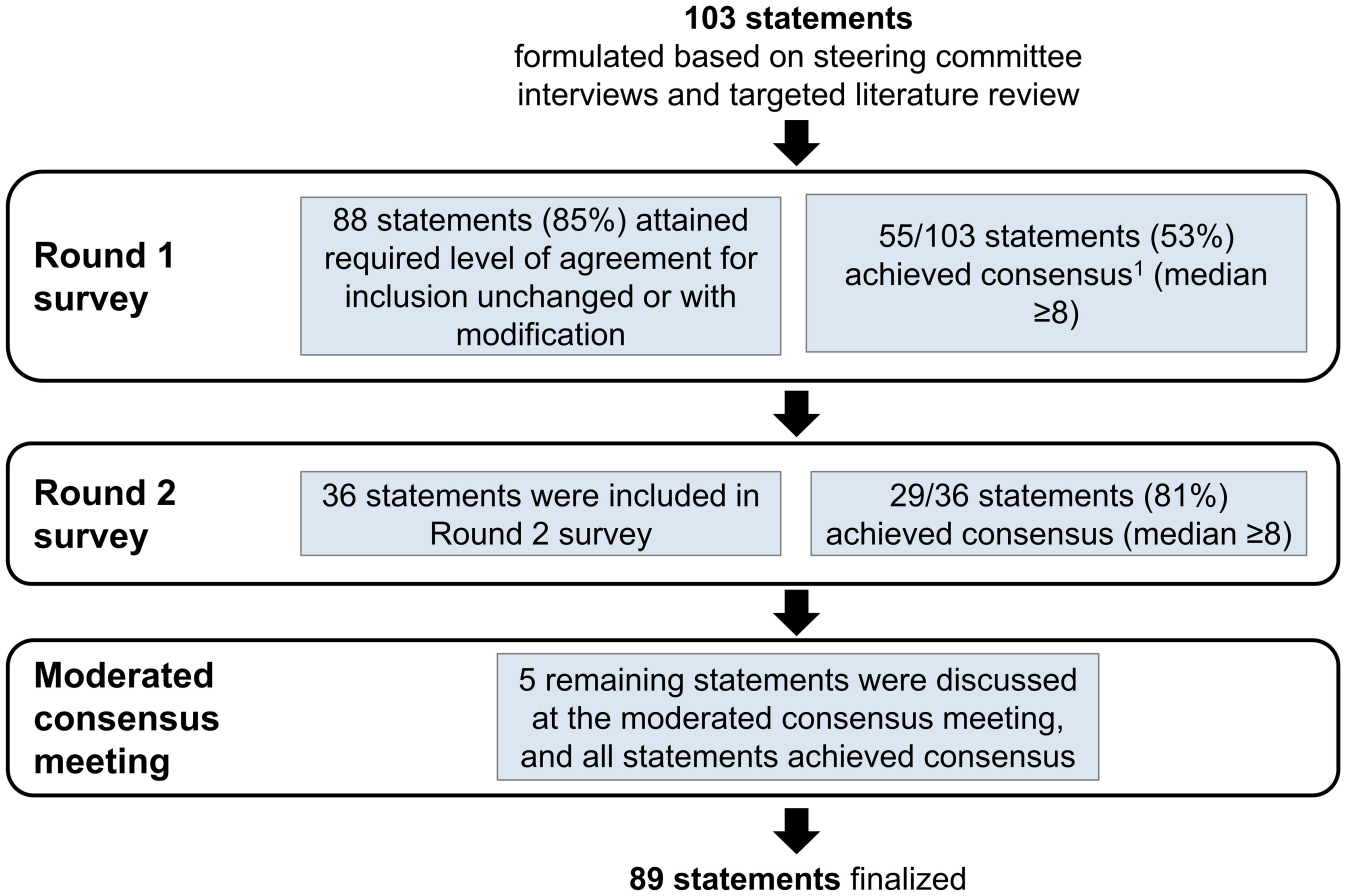
Formulation and refinement of consensus statements.

A total of 103 statements were presented to the panelists for scoring. Overall, consensus was achieved (median ≥8) on 89 statements (86%). About half of the statements (55/103, 53%) achieved consensus in Round 1 (**Figure 2**). In Round 2, a further 29 statements achieved consensus, out of 36 modified statements presented to the panelists for scoring (29/36). At the moderated consensus meeting, five modified statements were discussed by the steering committee and consensus was achieved on all statements (5/5). The statements are summarized according to the following thematic areas: clinical definitions and concepts (**Supplementary Table 3**), prevalence (**Supplementary Table 4**), risk factors (**Supplementary Table 5**), patient impact and clinical burden (**Supplementary Table 6**), diagnosis (**Supplementary Table 7**), treatment (**Supplementary Table 8**), and physician perspectives on novel therapies (**Supplementary Table 9**). The key insights from this study are presented in **Table 2**.

**Table 2:** Key insights from the modified Delphi panel study on MDDwA.

		Insights
Clinical definitions and concepts	1	Anhedonia is multifaceted and thus, the current definition in the DSM-5 that focuses only on motivational and consummatory anhedonia, is insufficient.
	2	Anhedonia is insufficiently recognized.
	3	There is clinical value and importance in defining MDDwA as a distinct mood subtype if there are: • Therapeutic options available that target anhedonia • Different treatment implications compared to other MDD subtypes • Robust evidence that MDDwA has a distinct and stable descriptive psychopathology
	4	MDDwA is not equivalent to MDDwM, but there may be some overlap between the two subtypes.
	5	Further research is needed to assess whether MDDwA has different psychopathology and treatment implications from other subtypes.
Prevalence and Risk factors	6	Factors contributing to variation in estimated prevalence across clinical populations include: • Culture and linguistics • Awareness of anhedonia • Inconsistencies in the definition of anhedonia
	7	MDDwA can affect individuals from all backgrounds and age ranges.
	8	Further research is needed to identify specific risk factors and support their association with MDDwA.
Diagnosis	9	Anhedonia is important to diagnose but can be challenging to detect and/or may be overlooked.
	10	Challenges in diagnosis of anhedonia include: • A lack of established diagnostic criteria & interview frameworks that are specific to anhedonia. • Assessment of anhedonia is subjective in nature. • Poor physician awareness of assessment scales to measure anhedonia.
	11	The severity of anhedonia is dependent on the clinician’s impressions & symptoms reported by patients.
	12	Assessment scales can be helpful for evaluating anhedonia but are mostly used in research.
	13	The SHAPS is potentially a suitable anhedonia assessment scale but its utilization among psychiatrists may vary.
	14	The DARS is an alternative to the SHAPS but less widely used.
	15	There is a need to develop simple and quick tools for the assessment of anhedonia.
Patient impact and clinical burden	16	Anhedonia in MDD patients is associated with poor overall clinical outcomes.
	17	The persistence of anhedonia is associated with poorer psychosocial functioning.
	18	The severity of anhedonia influences the disease course and extent of psychosocial impairment.
	19	Further research is required to understand clinical burden and patient impact of anhedonia, which negatively affects social relationships and leads to productivity losses.
Treatments	20	Treatment of anhedonia is important but limited by the availability of effective pharmacological agents and established guidelines.
	21	Differences in treatment choices across severities for MDDwA were observed across APAC countries/territories.
	22	It can be difficult to distinguish between emotional blunting and anhedonia
	23	Approaches to the management of emotional blunting vary across the medical community.
	24	Management of MDDwA can be optimized by enhancing patient engagement and perceived treatment value.
	25	Further research is required to assess the efficacy of various pharmacological treatments on anhedonia outcomes in MDD.
Physician perspectives of a novel therapy	26	An ideal therapeutic drug for MDDwA should be effective, safe and tolerable.
	27	Consensus surrounding potential comparators for a novel pharmacotherapy was not achieved.

### 3.1 Clinical definitions and concepts

The statements that achieved consensus in this thematic area are summarized in **Supplementary Table 3**. Most panelists agreed with the existing DSM-5 definition of anhedonia (second criteria A-2) as markedly diminished interest or pleasure in daily activities (7), and considered anhedonia a core feature of MDD (S1 and S2). However, the panelists concurred that anhedonia is multifaceted and there are still inconsistencies in the definition of anhedonia in MDD used amongst the medical community (S3). The panelists highlighted that the definition in the DSM-5 insufficiently reflects advances in the understanding of anhedonia (9, 10, 24). The panel also agreed that the definition of anhedonia in MDD should be expanded to encompass reduction in hedonic function including loss of interest and satisfaction in activities previously considered pleasurable and diminished motivation and desire to pursue pleasure or activities that generate pleasure (S4). Other facets of hedonic function were presented for consideration, but these did not achieve consensus (i.e., only half of the panelists [50%] selected the options of ‘paralysis of the sense of wellbeing’ and ‘paralysis of feeling’). Notably, the panelists agree that anhedonia is insufficiently recognized due to a lack of physician awareness of the nature, importance and impact of anhedonia (S5). One expert pointed out that although anhedonia is listed in the DSM-5 as a core diagnostic criterion, the lack of explicit mention of anhedonia symptoms could contribute to its insufficient recognition relative to other symptoms that are clearly listed.

### 3.2 Prevalence and risk factors

The statements that achieved consensus in these thematic areas are summarized in **Supplementary Tables 4** and **5**. There was consensus that anhedonia is one of the most frequent symptoms of depression (S16) and most panelists estimated that at least 40% of their MDD patients present with anhedonia, albeit with a wide range of estimates (S17; **Table 3**). This is consistent with published literature wherein estimates range from around 35–70% (10, 11). In the consensus meeting, one proposed explanation for the wide variation across countries/territories in estimated anhedonia prevalence was differences in the level of awareness and understanding of anhedonia, which could be in part due to differences in language and culture. For example, in Japan, the lack of a direct translation of the term “anhedonia” in Japanese and connotations of sexual anhedonia may result in under-reporting by patients. It was suggested that, if physicians in general were more aware of anhedonia in MDD, they would routinely ask their patients targeted questions specific to the various facets of anhedonia during consultations. The contribution of insufficient patient awareness to under-reporting of incidence of anhedonia in MDD patients was also discussed. For example, in Hong Kong, local mental health promotion activities largely focus on low mood, suicidal ideation and functional performance in MDD, overlooking anhedonia; this can lead to under-reporting due to low awareness of anhedonia. In summary, inconsistencies in definitions and diagnostic criteria for anhedonia may contribute to the variable prevalence of anhedonia estimated within the Asia-Pacific region.

**Table 3:** Estimated prevalence of MDDwA across the Asia-Pacific region.

Country/territory	Panelist 1 estimate	Panelist 2 estimate		Averaged estimate
Australia	40–50%	90%		68%
China	75%	70%		73%
Japan	50%	60%		55%
Hong Kong	75%	40%		58%
South Korea	30–40%	30–40%		35%
Taiwan	80%	30%		55%
Overall				57%

MDDwA, major depressive disorder with anhedonia.

In the consensus meeting, it was agreed that MDDwA affects individuals from all backgrounds and age ranges, and risk factors can include family history of depression, chronic health stressors and life events (S18, S19 and S21). However, it was noted that many of these are general risk factors for MDD and literature on identifying specific risk factors with MDDwA is scarce; thus, it was concluded that further research is needed to identify risk factors specific to MDDwA (S22).

### 3.3 Clinical impact and burden

The statements that achieved consensus in this thematic area are summarized in **Supplementary Table 6**. The panelists concurred that anhedonia is associated with poor overall clinical outcomes in MDD patients (S49 to S52). The panelists also agreed that anhedonia severity influences the disease course and extent of psychosocial impairment (S53, S59, and S61). This is in line with reported literature that more severe anhedonia is associated with the extent of depressive symptoms, illness chronicity and reduced psychosocial functioning (9, 11–13, 25). MDD patients with anhedonia may have an inherently higher likelihood of treatment non-adherence, due to deficits in motivation to engage in activities such as treatments and to anticipate positive outcomes [S54] (13). The panelists agreed that patients with anhedonia were more likely to stop their treatments prematurely.

### 3.4 Diagnosis

The statements that achieved consensus in this thematic area are summarized in **Supplementary Table 7**. Panelists agreed that anhedonia is challenging to detect and/or may be overlooked during diagnosis (S23 and S25). Several barriers to accurate diagnosis were highlighted.

First, established diagnostic criteria and interview frameworks that are specific to anhedonia are lacking (S24). The panel highlighted that although the DSM-5 provides some guidance, this is markedly limited and establishing well-defined criteria can guide diagnosis of anhedonia in clinical practice. A targeted literature review conducted as part of the study identified no clinical guidelines specific to anhedonia in MDD (**Supplementary Table 1**).

Second, anhedonia assessment is subjective, depending on both the physician’s skill and awareness, and the patient’s ability to articulate their feelings and/or experiences. Panelists agreed that diagnosis is based on good patient history taking by the physician, asking specific and targeted questions about anhedonia symptoms, and being perceptive to notice discrepancies between patients’ reported symptoms and behavior/signs elicited during interviews (S37 and S39). For patients, their ability to articulate their feelings and/or subjective experiences strongly influences whether anhedonia is identified during consultation (S40), as well as the physician’s impressions of anhedonia severity (S43).

Third, although a number of anhedonia assessment scales are available (e.g., SHAPS, DARS), the panelists agreed that the use of these is mostly confined to research (S28 to S30). They are not often used clinically, and physician awareness of these scales is poor. The SHAPS (a 14-item, self-reported questionnaire specifically assessing hedonic capacity) has been considered the gold standard for assessment of anhedonia (17). The SHAPS assesses anhedonia across four consummatory pleasure domains (hobbies/pastimes, food/drinks, social activities, and sensory experiences) using examples, but lacks assessment of anticipatory pleasure (S48). The DARS is a ‘second generation’ self-report scale designed to assess anhedonia across additional dimensions to pleasure (reward, interest, motivation, effort and pleasure) identified through principal component analysis (18). The 17 items include the same four pleasure domains as SHAPS but were designed to increase generalizability by asking respondents to provide their own examples for rating. Although the DARS is a more comprehensive scale to measure anhedonia, most of the panelists (up to 92%) were insufficiently familiar with it, and it is less widely used in clinical practice than the SHAPS (S45 to S47). Notably, despite high generalizability reported within western populations (18, 26–29), the SHAPS and DARS may lack cultural validity, particularly for Asian populations. In view of the significant variation in the understanding and expression of emotions across different cultures (30), non-English versions of the DARS (Chinese, German, Polish and Spanish) have been constructed and validated for use (31–34). The challenges described above may be compounded by the varied clinical definitions of anhedonia in use (8, 35).

A major limitation of the SHAPS and DARS highlighted by the panelists is the time taken for completion. Since anhedonia is a transdiagnostic symptom dimension present in multiple conditions (e.g., MDD, schizophrenia, bipolar disorder), comprehensive assessment scales such as the HAM-D (Hamilton Depression Rating Scale) and MADRS (Montgomery-Åsberg Depression Rating Scale) were seen as offering more time-effective alternatives as they measure a wider range of symptoms within a single test (S31). Overwhelmingly, panelists reported that they currently use MADRS (92%) and HAM-D (83%) when diagnosing anhedonia in MDD; only 42% use SHAPS and 25% use DARS. These findings emphasize the need to develop simple and quick tools for anhedonia assessment for clinical practice (S26) that measure the different facets of anhedonia, distinguish anhedonia from related constructs, and are generalizable for use across different cultures and populations (S27).

### 3.5 Treatment and clinical management

The statements that achieved consensus in this thematic area are summarized in **Supplementary Table 8**. Although the panelists agreed that it is important to treat anhedonia (S67), one of the challenges in treatment is the limited availability of effective pharmacotherapies and the lack of standardized, established management guidelines specific to anhedonia in MDD patients (S68 and S69). There are no pharmacotherapies specifically approved for treatment of anhedonia in MDD patients, although some agents (e.g., vortioxetine, agomelatine, bupropion) have been used (S71). In the consensus meeting, it was highlighted that selecting appropriate treatments based on the mechanism of action of various facets of anhedonia present in the patient can aid in predicting and improving treatment outcomes. Interestingly, a recent systematic review of 17 clinical studies concluded that most pharmacotherapeutic agents for MDD were associated with improvement in measures of anhedonia to varying degrees, except for escitalopram/riluzole combination treatment (36). However, due to the small number of studies and the heterogeneity of study designs and samples across the studies, further research is required to confirm these findings.

The panel’s responses indicate that treatment options in use across the Asia-Pacific region vary considerably, especially with respect to anhedonia severity. This is because prescribing choices are determined by local regulatory approval status and availability, cost/reimbursement policies, and national or regional MDD management guidelines and habits in clinical practice to some degree (37). Consensus was not achieved on potential treatment options for MDD patients with mild anhedonia, although these may include bupropion (150–300 mg), if available, and adjunctive cognitive behavioral therapy (S82). However, consensus was reached on potential treatment options for moderate and severe anhedonia in MDD patients. The panel concurred that treatment options for moderate anhedonia in MDD may include adjunctive aripiprazole (5–10 mg), adjunctive CBT and adjunctive rTMS (S83); and may include adjunctive aripiprazole, adjunctive mood stabilizers (e.g., lithium), adjunctive CBT and adjunctive rTMS for treatment of severe anhedonia in MDD (S84). Additionally, the panel agreed that third-line management options to treat severe anhedonia in MDD include adjunctive ECT (S85). Although a general consensus on treatment options for anhedonia in MDD patients was not achieved, it was agreed that management of MDDwA can be optimized through patient engagement and enhancing patients’ perception of the value of treatment (S86 to S89). Further research is required to assess the efficacy of various pharmacological treatments for anhedonia in MDD patients (S72 and S73).

Emotional blunting, defined as the inability to experience both positive and negative emotions, is present in several psychiatric disorders, and is described as a common side effect of antidepressants, particularly SSRIs, in MDD patients. Approximately half of MDD patients taking monoaminergic antidepressants reported experiencing emotional blunting [S76 (38–41);]. Over half of the panelists (58%) estimated that 30–49% of patients treated with SSRIs/SNRIs report some degree of emotional numbness or blunting. There was consensus that chronic SSRI administration may induce emotional blunting as a side effect (S80). Notably, this has implications for evaluating the effectiveness of SSRIs for treating MDD if anhedonia is present. It may be difficult to distinguish between emotional blunting as a side effect and residual anhedonia after treatment, and additional time and evaluation may be required to differentiate between the two (S74 and S75). As some patients may experience emotional blunting with SSRIs, whereas others may benefit from treatment without experiencing this side effect, there is still debate about the efficacy of SSRIs in treating anhedonia in MDD (S76). For patients experiencing emotional blunting on SSRI therapy, switching to a different agent may be an option, as observed in a recent study in MDD patients that showed positive effects on emotional blunting and other MDD symptoms after switching to vortioxetine (42).

Patients with MDD often have one or more physical or psychiatric comorbidities, such as cardiometabolic disorders, chronic pain-related conditions, or substance addiction, which may be associated with greater MDD severity and worse treatment outcomes [reviewed in (43–45)]. For example, MDD patients with higher insulin resistance showed more pronounced depressive symptomatology, including anhedonia, than those with lower insulin resistance (46). For several conditions, there is also considerable evidence of bidirectional effects [reviewed in (47)], underscoring the importance of managing both MDD/anhedonia and any comorbidities. The presence of comorbid conditions also increases the complexity and challenges of clinical management, such as communication/coordination between treating physicians and teams to manage each condition, and greater attention to drug-drug interactions and adverse side effects from polypharmacy.

### 3.6 Physician perspectives on novel therapies for MDDwA

The statements that achieved consensus in this thematic area are summarized in **Supplementary Table 9**. An ideal novel pharmacotherapy for MDDwA should effectively target anhedonia alongside other MDD symptoms, support physical, social, cognitive, and functional recovery, and should have a good safety and tolerability profile (S90). Although consensus was not achieved on suitable comparators for head-to-head trials of novel pharmacotherapy agents, a number of commonly prescribed agents (e.g., monotherapy escitalopram, adjunctive intranasal esketamine, monotherapy agomelatine, monotherapy vortioxetine, adjunctive aripiprazole and adjunctive bupropion) were mentioned as potentially useful comparators (S92). For example, a recent pooled analysis reported that agomelatine and vortioxetine showed significant short-term efficacy for anhedonia (48). The experts’ feedback emphasized that novel pharmacotherapies should demonstrate superiority for anhedonia and similar efficacy for MDD overall, with respect to existing treatment options (S92).

### 3.7 MDDwA as a distinct subtype of depression

Importantly, there was clearly perceived clinical value in establishing MDDwA as a distinct MDD subtype to optimize treatment/management and to develop targeted treatments that may increase response and remission rates (S8). Establishing MDDwA as a distinct subtype was considered reasonable if: there was robust evidence for a distinct and stable descriptive psychopathology; treatment implications differ from other MDD subtypes; and type-specific therapeutic options are available (S9).

Most of the panel (82%) supported the establishment of MDDwA as a distinct subtype to inform treatment decision-making and identification of subgroups, such as the choice of treatment if anhedonia is present (S12). The current challenge in delineating MDDwA as a distinct subtype may lie in distinguishing it from MDD with melancholia (MDDwM), since anhedonia has been proposed as one of the markers of MDDwM (49). On the other hand, recent data-driven approaches to define MDD subtypes using neurobiological and clinical features [reviewed in (50)] point to the relevance of anhedonia in defining MDD subtypes, besides being a core diagnostic feature. In one study, patterns of functional network connectivity and clinical symptoms were used to identify two putative subtypes, an insomnia-dominated subtype and an anhedonia-dominated subtype (21). Another study identified four putative subtypes with distinct patterns of functional connectivity, each associated with specific profiles of clinical symptoms. Of these four subtypes, two were characterized by increased anhedonia and psychomotor retardation, and two by increased anxiety and fatigue (51). The prevailing view among the panelists was that MDDwA differs from MDDwM in several ways (S13). MDDwM has a distinct and characteristic symptom cluster (i.e., generalized psychomotor retardation, weight loss/loss of appetite, early morning waking, excessive guilt, sense of hopelessness and diurnal variation (7)) that is absent in MDDwA. Patients with MDDwM tend to present with more severe affect and symptoms along with difficulty in mood elevation, compared with MDD patients with anhedonia. There are also reported differences in the brain areas involved, mainly the frontal area for MDDwM patients, and reward-related brain areas (e.g., insula, anterior cingulate cortex, orbitofrontal cortex) in MDD patients with anhedonia (S13).

### 3.8 Strengths and limitations of this work

The modified Delphi process provided a suitable means of exploring this complex topic, given the diverse models and conceptualizations of anhedonia in MDD, lack of gold-standard anhedonia-specific practice guidelines, and variability in clinical practice. A targeted literature research also revealed a paucity of research and quantitative data for the region, reinforcing the value of an expert panel for generating insights. Another strength of this study is expert representation from countries/territories across the Asia-Pacific region within both the steering committee and the panelists. This allowed variations in local diagnostic and treatment practice to be captured, as well as providing insights into potential reasons for the wide range of estimated prevalence of anhedonia across countries/territories.

One limitation of the study is the relatively small number of panelists (n=12) included. Researchers have proposed optimum panel sizes ranging from 10 to 50, while also acknowledging that this number depends on factors such as the target topic, degree of panel homogeneity, the expected amount of data generated per participant, and the resources available (52, 53). In addition to the data generated from the 12 expert panelists’ responses, it was considered that the involvement of a steering committee comprising six additional experts would enable adequate alignment on the consensus statements and non-consensus insights retained for discussion. We acknowledge that a larger and more diverse panel of practitioners, perhaps representing additional countries/territories within the Asia-Pacific, as well as a greater range of practice settings, would better capture the variation that exists in health systems.

### 3.9 Future work

The present work underscores the lack of clear and practical methods in clinical practice for assessing anhedonia in MDD patients, with implications for both diagnosis and management. Within the Asia-Pacific region, poor physician awareness of anhedonia was identified as a prominent unmet need. With a variable but likely underestimated prevalence of anhedonia in MDD patients (35–70% as estimated by panelists), attention and awareness among both physicians and patients is urgently warranted. The panel agreed that further research is needed to clarify and refine the definitions and concepts in use and distinguish anhedonia from other symptom domains in MDD. This would be complemented by ongoing efforts to elucidate the neurobiological mechanisms underlying anhedonia. Within the region, initiatives to assess anhedonia prevalence in a more standardized way and quantify its impact on treatment adherence and outcomes could help promote awareness among physicians and patients, and potentially reduce delays in diagnosis and treatment. One limitation is the lack of availability of quantitative data in the region and hence, future work should prioritize quantitative studies of the prevalence and impact of anhedonia in MDD patients across the region. We also note that, in a culturally diverse region such as the Asia Pacific, educational initiatives for physicians and patients should be developed with the local sociocultural context and patient population in mind. All of these initiatives must be supported by efforts to evaluate and develop treatment options with greater efficacy specifically for anhedonia than existing treatments but similar efficacy for MDD overall, and with less likelihood of side-effects such as emotional blunting.

## 4 Conclusion

Identification of distinct and clinically relevant subtypes in MDD may be valuable for guiding personalized diagnosis and management approaches, including type-specific therapies. Given the importance of anhedonia in MDD diagnosis, prognosis, and treatment decision-making, recognizing a distinct depressive subtype characterized by anhedonia could help improve clinical management and guide the development of new treatment options. While acknowledging that considerable conceptual and mechanistic research is still needed, the study highlighted a fundamental need for simple and accurate tools to assess anhedonia in routine practice settings. Insights from this Delphi consensus provide a reference point for psychiatrists in the Asia-Pacific to optimize their strategies for personalized diagnosis and management of patients with MDDwA.

## Data availability statement

The original contributions presented in the study are included in the article/**Supplementary Material**. Further inquiries can be directed to the corresponding author.

## Author contributions

CC: Writing – review & editing, Conceptualization. KH: Writing – review & editing, Conceptualization, Methodology. HJ: Conceptualization, Writing – review & editing. TK: Conceptualization, Writing – review & editing. CN: Conceptualization, Writing – review & editing. YY: Conceptualization, Writing – review & editing. LZ: Conceptualization, Writing – review & editing.
