# Supplementary material for: A Delphi consensus on clinical features, diagnosis and treatment of major depressive disorder patients with anhedonia amongst psychiatrists in the Asia-Pacific
Source: Front Psychiatry. 2024 Feb 23;15:1338063. doi: 10.3389/fpsyt.2024.1338063 (PMC10920342; doi:10.3389/fpsyt.2024.1338063)
Supplement: Supplementary file 1 [file Table_1.docx]

# Supplementary Table 1. Targeted literature review strategy

| **Search Strategy** | |
| --- | --- |
| Database | PubMed |
| Date | 18 Oct 2022 |
| Search terms | Query #1:”depressive disorder, major”[MeSH Terms] OR major depression[Title/Abstract] OR major depressive disorder[Title/Abstract] NOT “bipolar and related disorders”[MeSH Terms] Filters: in the last 10 years, Humans  Query #2:”antidepressive agents”[MeSH Terms] OR antidepressive[Title/Abstract] OR antidepressant[Title/Abstract] OR pharmacological[Title/Abstract] OR “cognitive behavioral therapy”[MeSH Terms] OR “psychotherapy”[MeSH Terms] OR non-pharmacological[Title/Abstract] Filters: in the last 10 years, Humans  Query #3: “anhedonia”[MeSH Terms] OR lack of pleasure[Title/Abstract] OR lack of interest[Title/Abstract] OR lack of motivation[Title/Abstract] NOT “anhedonia/physiology”[MeSH Terms] Filters: in the last 10 years, Humans  (#1 OR #2) AND #3 Filters: in the last 10 years, Humans |
| Period | 2012 to 2022 |
| Country | Global |
| Language | English |
| Species | Restricted to Humans |
| Hits | 281 |

# Supplementary Table 2. Targeted literature review output

| **Search output** | |
| --- | --- |
| Number of search hits | 281 |
| After title screening | 169 |
| After abstract screening | 78 |
| After full-text screening | 31 |
| Final number of articles reviewed | Total: 35  31 from PubMed search  4 additional publications^1^ |
| **Summary of outputs mapped to themes^2^** | |
| Clinical definition | 35 |
| Diagnosis | 31 |
| Disease burden | 30 |
| Risk factors | 10 |
| Treatment and management | 20 |
| Clinical guidelines | 0 |

^1^ Additional publications were not directly captured by search strategy, but were added upon advice as they were aligned with the defined search criteria

^2^ Number of articles do not add up to 35 as some articles address more than one theme.

# Supplementary Table 3. Final list of recommendations – Clinical definitions and concepts

| **#** | **Question/statement** | **Median (IQR) / % rated as 7–9^1^** | **Consensus achieved in** |
| --- | --- | --- | --- |
| S1 | Anhedonia is defined as that in the DSM-5, which specifies anhedonia as “diminished interest [motivational anhedonia] or pleasure [consummatory anhedonia].” | 8 (1.25) | R2 |
| S2 | Traditionally, anhedonia is considered a core feature of MDD. | 9 (1.00) | R1 |
| S3 | Anhedonia is multifaceted and can be defined as:   - Inability to experience pleasure in activities previously considered pleasurable - Impaired ability to pursue pleasure or activities that generate pleasure - Reduced reward anticipation - Reduced or loss of emotional reactivity to reward or pleasurable stimuli   However, there are still perceived inconsistencies in the definition of anhedonia in MDD amongst the medical community | 9 (1.00) | R2 |
| S4 | Additionally, the following facets of hedonic function should also be considered in the definition of anhedonia, within the context of MDD:   - Loss of interest and satisfaction in activities previously considered pleasurable - Diminished motivation and desire to pursue pleasure or activities that generate pleasure | 8 (1.25) | R2 |
| S5 | Amongst the medical community, there is insufficient recognition given to anhedonia in the context of MDD due to a lack of physician awareness of the nature, importance, and impact of anhedonia | 8.5 (2.25) | R1 |
| S6 | Anhedonia may exist on its own as a distinct feature that is independent from MDD. | 8 (1.5) | R2 |
| S7 | The signs and symptoms of anhedonia, in the context of MDD, can occur often and in daily life. | 8 (1.25) | R2 |
| S8 | Overall, there is clinical value and importance in defining MDDwA as a distinct mood subtype to:   - Optimize pharmacological and non-pharmacological management - Develop targeted treatments which may increase response and remission rates | 83%  83% | R1 |
| S9 | There is clinical value and importance in defining MDDwA as a mood subtype to:   - Aid physician awareness and understanding to facilitate accurate and standardized diagnosis - Identify patient subgroups with similar characteristics who may preferentially benefit from available treatment   If there is / are:   - Therapeutic options available which target anhedonia; - Different treatment implications compared to other MDD subtypes; and - Robust evidence that MDDwA has a distinct and stable descriptive psycho-pathology | 8 (1.25) | R2 |
| S10 | Though anhedonia is considered a basic trait for depression, MDDwA is not equivalent to the melancholic subtype. | 8.5 (2) | R1 |
| S11 | Further research is needed to determine how and whether MDDwA can be distinguished from MDDwM. | 9 (0.75) | CM |
| S12 | The DSM-5 does not recognize MDDwA as a mood subtype and positions anhedonia as a core depressive symptom that forms part of diagnostic criteria for MDD; majority of the panel (82%) supported the establishment of MDDwA as a distinct subtype as this can potentially:   - Inform treatment decision-making – there is some evidence that anhedonia can influence choice of treatment specifically (e.g., poorer response to SSRIs relative to non-anhedonic MDD); and - Help to identify subgroups with certain characteristics (e.g., presentation, prognostic factors, etc.), particularly for patients who present with anhedonia but do not meet the standard definition of MDD   Further research is however required to explore and evaluate MDDwA as a potential subtype with different, distinct and stable descriptive psychopathology and different treatment implications compared to other MDD subtypes.   - Within the context of MDD, patients often present with anhedonia; if the new subtype is to be distinguished, then it needs to be clearly segregated from other MDD subtypes, particularly MDD with melancholia - While a broader definition may aid in more effective symptom recognition, it may promote a vague understanding of MDD and impede the progression of MDD therapy research. | N/A (free text) | R1 |
| S13 | Key differentiators between MDDwA and MDD with melancholia (MDDwM) were:   - MDDwM is a subtype that has several key symptoms which are not necessarily seen in MDDwA, including ortioxetin psychomotor retardation, weight loss / loss of appetite, early morning waking, excessive guilt, a sense of hopelessness / helplessness and diurnal variation. - MDDwM patients tend to present with more severe affect and symptoms, alongside difficulties with mood elevation. In contrast, anhedonia is mostly seen in treatment resistant MDD patients and they often respond to changes in circumstance and retain some desire / motivation (but unable to feel pleasure). - Neurobiologically, anhedonia is related to reward-related brain areas, e.g., insula, anterior cingulate cortex, orbitofrontal cortex, whereas MDDwM is concentrated to the frontal area. | N/A (free text) | R1 |
| S14 | There should be greater academic research focus on defining, and distinguishing anhedonia from other symptom domains in MDD. | 9 (1.25) | R1 |
| S15 | There should be greater academic research focus on assessing the humanistic burden of anhedonia in patients with MDD (i.e., distinct from clinical burden, and encompasses patient’s functional recovery, and Quality of Life). | 9 (1.5) | R1 |

^1^ If there were any discrepancies between the % rating and the median score, the median score was used to determine level of consensus. The % rating was used to guide analysis and statement modification.

R1, Round 1; R2, Round 2; CM: consensus meeting

# Supplementary Table 4. Final list of recommendations – Prevalence

| **#** | **Question/statement** | **Median (IQR) / % rated as 7–9^1^** | **Consensus achieved in** |
| --- | --- | --- | --- |
| S16 | Anhedonia, is one of the most frequent symptoms of depression. | 8 (3) | R1 |
| S17 | Majority of the panel estimated that at least 40% of MDD patients presented with anhedonia, however, inter- and intra-market variabilities in prevalence of anhedonia amongst MDD patients were observed.   \|  \| **Panelist 1** \| **Panelist 2** \| **Average** \| \| --- \| --- \| --- \| --- \| \| **AU** \| 40–50% \| 90% \| 67.5% \| \| **CN** \| 75% \| 70% \| 72.5% \| \| **JP** \| 50% \| 60% \| 55% \| \| **HK** \| 75% \| 40% \| 57.5% \| \| **SK** \| 30–40% \| 30–40% \| 35% \| \| **TW** \| 80% \| 30% \| 55% \| \| **Regional average** \| \| \| 57% (~60%) \| | N/A (free text) | R1 |

^1^ If there were any discrepancies between the % rating and the median score, the median score was used to determine level of consensus. The % rating was used to guide analysis and statement modification.

R1, Round 1; R2, Round 2; CM: consensus meeting

# Supplementary Table 5. Final list of recommendations – Risk factors

| **#** | **Question/statement** | **Median (IQR) / % rated as 7–9^1^** | **Consensus achieved in** |
| --- | --- | --- | --- |
| S18 | MDDwA can affect people from all backgrounds, i.e. not specific to any ethnicity, socioeconomic or marital status. | 9 (1) | R1 |
| S19 | The following can be risk factors for patients with MDDwA:   - Family history of any type of depression - Chronic health stressors, i.e., chronic illnesses such as inflammatory conditions, diabetes, heart disease and cancer - Life events, e.g., bereavement, employment, divorce, social conflict | 83%  92%  100% | R1 |
| S20 | Patients who have moderate to severe MDDwA tend to present with social occupational impairment, e.g., in family, work, studies | 9 (0.25) | R1 |
| S21 | MDDwA can affect individuals of all ages and the age of onset can vary widely | 8 (0.75) | CM |
| S22 | There is a variety of potential risk factors for MDDwA and further research is needed to identify specific risk factors. | 9 (0.75) | CM |

^1^ If there were any discrepancies between the % rating and the median score, the median score was used to determine level of consensus. The % rating was used to guide analysis and statement modification.

R1, Round 1; R2, Round 2; CM: consensus meeting

# Supplementary Table 6. Final list of recommendations – Patient impact and clinical burden

| **#** | **Question/statement** | **Median (IQR) / % rated as 7–9^1^** | **Consensus achieved in** |
| --- | --- | --- | --- |
| S49 | Anhedonia in the context of MDD is associated with and has an impact on patients’ social functioning. | 9 (1) | R1 |
| S50 | Anhedonia, rather than depressed mood, has a greater and more negative impact on psychosocial functioning. | 8 (2) | R1 |
| S51 | In the context of MDD, anhedonia is associated with:   - Lower remission rates - Poorer prognosis and worse outcomes - Non-response or resistance to pharmacotherapy and psychotherapy - Greater functional impact - Impaired individual Quality of Life - Impaired caregiver Quality of Life - High rates of social withdrawal - Poorer productivity (e.g., work, school) | 92%  100%  100%  92%  100%  92%  92%  100% | R1 |
| S52 | In the context of MDD, anhedonia is associated with a longer time to remission. | 92% | R2 |
| S53 | Improvements in anhedonic symptoms are associated with improvements in self-reported psychosocial functioning. | 8(2) | R1 |
| S54 | In the context of MDD, anhedonia may interfere with treatment engagement and adherence (due to inherent motivation deficits to engage in activities and anticipate positive consequences). | 8.5 (1) | R1 |
| S55 | Anhedonia is a symptomatic barrier to functional recovery in MDD. | 8.5 (1.25) | R1 |
| S56 | In the context of MDD, the persistence of anhedonia is associated with poorer psychosocial functioning. | 8 (2) | R2 |
| S57 | In the context of MDD, anhedonia may persist as a residual symptom and can be complicated by treatment-related emotional blunting. | 8.5 (1.25) | R2 |
| S58 | In the context of MDD, the persistence of anhedonia as a residual symptom may be overlooked, particularly when other symptom domains of MDD have improved. | 8 (0.5) | R2 |
| S59 | In the context of MDD, anhedonia can exist on a spectrum, whereby mild anhedonia may involve self-observed, limited impairment to psychosocial functioning; in contrast, severe anhedonia significantly impairs individuals’ abilities to engage in activities of daily living. | 8.5 (1.25) | R2 |
| S60 | MDD patients diagnosed with severe anhedonia are more likely to experience a more chronic, treatment-resistant course of illness than their mild or moderate counterparts. | 8 (1.25) | R1 |
| S61 | In the context of MDD, the extent of psychosocial impairment is correlated with the severity of anhedonia, i.e., there is severe impact to psychosocial functioning in severe anhedonia. | 8 (2) | R2 |
| S62 | In the context of MDD, severe anhedonia is associated with an increased likelihood of relapse even when remission does occur. | 8 (2) | R1 |
| S63 | MDD patients with severe anhedonia are most in need of treatment but may have greater impairment in effort valuation, resulting in greater perception of barriers and fewer motivational resources to draw upon. | 8 (1) | R1 |
| S64 | It can be difficult for patients to verbalize and/or recognize anhedonic symptoms. | 8 (2.25) | R1 |
| S65 | Caregivers, family members and friends of MDDwA patients are more likely to observe the external implications of anhedonia (e.g., lack of day-to-day activity functioning, social withdrawal). | 8 (0.5) | R1 |
| S66 | In the context of MDD, anhedonia has a notable negative impact on the economy due to costs associated with functional impairment (e.g., absenteeism from work, reduced productivity). | 8.5 (1.25) | R1 |

^1^ If there were any discrepancies between the % rating and the median score, the median score was used to determine level of consensus. The % rating was used to guide analysis and statement modification.

R1, Round 1; R2, Round 2; CM: consensus meeting

# Supplementary Table 7. Final list of recommendations – Diagnosis

| **#** | **Question/statement** | **Median (IQR) / % rated as 7–9^1^** | **Consensus achieved in** |
| --- | --- | --- | --- |
| S23 | In patients with MDD, anhedonia is important to diagnose. | 8.5 (1) | R1 |
| S24 | There is a lack of diagnostic criteria and guidelines that are specific to anhedonia for MDD patients. | 8 (0) | R1 |
| S25 | In patients with MDD, anhedonia can be difficult to detect and / or overlooked during diagnosis. This may be due to:   - A lack of defined diagnosis criteria to assist clinicians with examining the whole range of anhedonic symptoms in clinical settings - A lack of standardized, objective measurements to assess severities of anhedonia - Limited consultation time (in some settings) - Anhedonia being masked in partially-treated patients - Patients not (or having difficulty) reporting anhedonic symptoms, particularly during subjective interviewing | 8 (2) | R2 |
| S26 | There is a need to develop simple and quick tools to assess anhedonia and psychosocial functioning in the context of MDD | 9 (1) | R1 |
| S27 | Ideally, a screening scale quantifying anhedonia in the context of MDD should be able to measure different aspects of anhedonia, detect state versus trait differences, distinguish anhedonia and related constructs, and be appropriate for use in samples with a range of cultural beliefs and preferences (i.e., generalizable). | 9 (1.25) | R1 |
| S28 | In routine clinical practice, assessment scales, e.g., Snaith Hamilton Pleasure Scale (SHAPS) and Dimensional Anhedonia Rating Scale (DARS), to measure and diagnose anhedonia in MDD patients are not frequently used. | 9 (0) | R1 |
| S29 | Assessment scales (i.e., SHAPS and DARS) are helpful for evaluating anhedonia over a period of time and changes in response to treatment. | 9 (1) | R1 |
| S30 | Assessment scales are mostly used in research contexts. | 8 (1.25) | R1 |
| S31 | HAM-D and MADRS were assessment scales most frequently adopted in clinical practice to specifically diagnose anhedonia in MDD patients.   - Montgomery–Åsberg Depression Rating Scale (MADRS) - Hamilton Depression Rating Scale (HAM-D) | 83%  92% | R1 |
| S32 | The SHAPS is considered the gold standard in terms of assessment scales for anhedonia in MDD due to its ability to question the experience of a definitive period instead of the continuous characteristics seen in a patient (i.e., measures state anhedonia) proven validity and reliability, and low cultural bias. | 8 (2) | R1 |
| S33 | Currently, the SHAPS is mostly used in clinical research; it is not widely adopted in clinical practice and there is variability in how familiar clinicians are with the use of the scale | 8 (1) | R2 |
| S34 | For the 4-point SHAPS scale, 50% reduction from baseline SHAPS score is used to define symptomatic response / improvement. | N/A (free text) | R1 |
| S35 | For the SHAPS, there is a need to establish clear, standardized cut-off values to measure levels of anhedonia in MDD, as well as validate across different cultures / populations. | 9 (1) | R2 |
| S36 | The SHAPS is a better tool for the assessment of anhedonia than the FCPS or CPAS / CSAS due to its wider applicability (i.e., minimal cultural bias), temporal considerations, and shorter time to complete. | 8 (1.5) | R1 |
| S37 | In routine clinical practice, anhedonia is primarily diagnosed via good patient history taking and specific targeted questioning of anhedonic symptoms in patients with MDD. | 8.5 (1) | R1 |
| S38 | The Mini International Neuropsychiatric Interview 5.0 (MINI) is a short diagnostic structured interview to diagnose MDD but is not specific to anhedonia. | 9 (1) | R1 |
| S39 | Accurate diagnosis of anhedonia in patients requires attention to differences between anhedonic symptoms (patients’ subjective complaints) and signs (behaviour deemed pathological or mood observed) elicited during a psychiatric interview. | 9 (1) | R1 |
| S40 | The ability for patients to coherently articulate what they find pleasurable or interesting is the greatest challenge in measuring anhedonia in MDD patients. | 8 (1.25) | R1 |
| S41 | The ability to accurately comprehend the subjective nature of what patients find pleasurable / interesting, as well as poor awareness of anhedonia scales, are some of the challenges that clinicians may face when measuring anhedonia in MDD patients. | 8 (0.25) | R2 |
| S42 | The differentiating symptoms across mild, moderate, and severe anhedonia in the context of MDD can be ortioxetin into:   \|  \| **Mild** \| **Moderate** \| **Severe** \| \| --- \| --- \| --- \| --- \| \| **Functional impairment** \| Limited to none \| Some \| Significant \| \| **Engagement in daily activities** \| 60–80% \| 25–50% \| 0–25% \| \| **Interest, enjoyment, pleasure and/or motivation** (*depending on how anhedonia is defined*) \| May experience some (though limited) loss in some previously pleasurable activities \| Increasing loss in a higher number of previously pleasurable activities, but some enjoyment is maintained \| Complete loss of consumptive & anticipatory pleasure and motivation in pursuing pleasurable activities (“completely going through the motions”) \| \| **Impact on relationships** \| Limited to no impact; mostly self-reported symptoms (e.g. cannot express affect) \| Some impact as family members / caregivers may be able to observe anhedonic symptoms \| Likely to significantly impact their closest relationships \| \| **Mood and energy** \| Limited to low mood \| May experience some fatigue \| Lack of energy to pursue / carry out activities and may “feel numb” \| \| **Others** \| N/A \| N/A \| Can experience emotional blunting and lack emotional responses to events that are usually influential; can present with limited words and reduced facial expressions \| | N/A (free text) | R1 |
| S43 | The classification of MDD patients into mild, moderate, and severe anhedonia is based on the clinician’s impressions and patients’ self-reported symptoms. | 8 (1.25) | R2 |
| S44 | Poor physician awareness is a key barrier to the uptake of assessment scales for the measurement of anhedonia in MDD. | 83% | R1 |
| S45 | Relative to the SHAPS, the DARS is less widely used in clinical practice.  Note: *5% unfamiliar with the SHAPS / DARS; Median and IQR calculations have excluded the response from a panelist who indicated that he does not have detailed knowledge about the SHAPS / DARS.* | 8 (1) | R1 |
| S46 | Up to 92% were unfamiliar with DARS; only 8% (1 panelist) was familiar. | N/A (free text) | R1 |
| S47 | Relative to the SHAPS, the DARS covers 4 components of reward processing (desire, motivation, effort and consummatory pleasure) and so may more comprehensively assess multidimensional features of anhedonia | 8 (1) | R2 |
| S48 | The SHAPS mainly assesses the consummatory pleasure (i.e., the momentary pleasure that is experienced while engaged in an enjoyable activity) of anhedonia rather than anticipatory pleasure (i.e., the expectation of pleasure related to future activities). | 8 (0.75) | CM |

^1^ If there were any discrepancies between the % rating and the median score, the median score was used to determine level of consensus. The % rating was used to guide analysis and statement modification.

R1, Round 1; R2, Round 2; CM: consensus meeting

# Supplementary Table 8. Final ist of recommendations – Treatment

| **#** | **Question/statement** | **Median (IQR) / % rated as 7–9^1^** | **Consensus achieved in** |
| --- | --- | --- | --- |
| S67 | In patients with MDD, anhedonia is important to treat. | 9 (1) | R1 |
| S68 | In patients with MDD, anhedonia can be difficult to treat, and this may be due to   - Poor physician awareness (relative to other symptom domains of MDD); - Lack of a clear and consistent definition; and/or - Limited pharmacological treatments available that effectively target anhedonic symptoms | 8 (1) | R2 |
| S69 | There is a lack of treatment guidelines specific to anhedonia in patients with MDD. | 8 (1) | R1 |
| S70 | Currently, available treatments for depression may improve anhedonia to a lesser extent than depressed mood. | 8 (2) | R1 |
| S71 | Currently, there are no specific pharmacological agents approved for the treatment of anhedonia for MDD patients, although some agents (e.g., vortioxetine, agomelatine, bupropion) have been used. | 8 (2) | R2 |
| S72 | Compared with other symptom domains, there is a relative paucity of studies evaluating anhedonia outcomes in MDD. | 8.5 (1.25) | R1 |
| S73 | Further research is needed to evaluate the comparative efficacy of different pharmacological agents on measures of anhedonia. | 9 (0) | R1 |
| S74 | In the context of MDD, anhedonia is the loss of ability to experience positive emotions. In contrast, emotional blunting is the loss of ability to experience both positive and negative emotions, where this perception is blunted and impaired. | 8 (0.5) | R1 |
| S75 | It can be difficult to distinguish emotional blunting from anhedonia in MDD in some patients; additional time and evaluation may be required to differentiate between the two. | 8 (1.25) | R2 |
| S76 | There is debate about the efficacy of SSRIs in treating anhedonia in MDD. Some patients may benefit from SSRIs without experiencing emotional blunting, while others may experience this side effect. | 8 (2) | R2 |
| S77 | Amongst the medical community, there is variability in how clinicians treat SSRI-induced emotional blunting; a common approach is (but not limited to) switching to another antidepressant class (e.g., vortioxetine, agomelatine, bupropion) or adding on an adjunctive antipsychotic (e.g., aripiprazole). | 8 (0) | R2 |
| S78 | Additionally, another approach could be to add or switch to non-pharmacological interventions (e.g. CBT and rTMS). | 8 (1) | R2 |
| S79 | In cases where SSRIs are not effective in improving positive affect, alternative anti-anhedonic treatments may be necessary to alleviate anhedonia in MDD. | 8 (1.25) | R2 |
| S80 | Chronic SSRI administration such as citalopram and fluoxetine may induce emotional blunting (i.e., The loss of ability to experience both positive and negative emotions, where this perception is blunted and impaired) as a side effect and can exacerbate levels of anhedonia in certain sub-populations. | 8 (2.5) | R1 |
| S81 | There was no consensus on the percentage of patients treated with SSRIs or SNRIs who report some degree of emotional numbness or blunting:   - <30% - 30% to 49% - 50% to 69% - ≥70% | 17%  58%  17%  8% | Consensus not achieved, retained as key insights |
| S82 | Treatment options for mild anhedonia in MDD may include (but are not limited to) monotherapy bupropion 150–300mg (if available in the market) and adjunctive CBT. | 7.5 (1) | Consensus not achieved, retained as key insights |
| S83 | Treatments options for moderate anhedonia in MDD may include (but are not limited to) adjunctive aripiprazole 5–10mg, adjunctive CBT and adjunctive rTMS. | 8 (1) | R2 |
| S84 | Treatments options for severe anhedonia in MDD may include (but are not limited to) adjunctive aripiprazole, adjunctive mood stabilizers, e.g., lithium, adjunctive CBT and adjunctive rTMS. | 8 (1) | R2 |
| S85 | Third-line management options to treat severe MDDwA include adjunctive ECT | 83% | R1 |
| S86 | Physicians can increase MDDwA patients’ perceived value of pharmacological or psychotherapeutic treatment by enhancing their perceptions of treatment value (e.g., providing accurate and realistic evidence for its efficacy, highlighting providers’ skills and competencies, and describing success examples). | 8 (1.25) | R1 |
| S87 | Physicians should implement approaches that seek to enhance anticipation of treatment and emphasize its potential benefits. | 9 (1) | R1 |
| S88 | Physicians should be encouraged to show positive emotions (e.g., warmth, empathy, and humor) with MDDwA patients. | 8.5 (3) | R1 |
| S89 | To help improve patient engagement and adherence towards a common treatment goal, some MDDwA patients may benefit from family, caregivers, friends and physicians defining positive moments in the patient’s life. | 8 (1.5) | CM |

^1^ If there were any discrepancies between the % rating and the median score, the median score was used to determine level of consensus. The % rating was used to guide analysis and statement modification.

R1, Round 1; R2, Round 2; CBT, cognitive behavioral therapy; CM: consensus meeting; rTMS, repetitive transcranial magnetic stimulation

# Supplementary Table 9. Final ist of recommendations – Physician perspectives on novel therapies

| **#** | **Question/statement** | **Median (IQR) / % rated as 7–9^1^** | **Consensus achieved in** |
| --- | --- | --- | --- |
| S90 | Ideally, a novel pharmacotherapy for MDDwA should have the following key attributes:   - Effectively target symptoms of both MDD and anhedonia; - Enable individuals to achieve physical, social, and cognitive functional recovery; - Have a good safety profile and be well-tolerated | 9 (0) | R2 |
| S91 | In addition, a good to have attribute for the novel pharmacotherapy would be a quicker onset of action relative to existing treatments. | 9 (1.25) | R2 |
| S92 | There was no consensus on potential clinical head-to-head comparators for a novel pharmacotherapy for MDDwA:  *The following pharmacological agents were selected by the panel:*   - Monotherapy escitalopram - Adjunctive intranasal esketamine - Monotherapy agomelatine - Monotherapy vortioxetine - Adjunctive aripiprazole - Adjunctive bupropion   *Panel feedback:*   - The majority indicated that widely prescribed therapeutic agents are useful comparators to demonstrate superiority for anhedonia and similar efficacy for MDD overall. - If the novel pharmacotherapy possesses multi-receptor targeting, it may be useful to choose an agent that does not overlap with receptors. - Additionally, agents that are less likely to cause emotional blunting as a side effect may be helpful, e.g., sertraline. | 33%  33%  25%  25%  25%  25% | Consensus not achieved, retained as key insights |

^1^ If there were any discrepancies between the % rating and the median score, the median score was used to determine level of consensus. The % rating was used to guide analysis and statement modification.

R1, Round 1; R2, Round 2; CM: consensus meeting
